# Supplementary material for: Identification of the Principle of Taste Sensors to Detect Non-Charged Bitter Substances by 1H-NMR Measurement
Source: Sensors (Basel). 2022 Mar 28;22(7):2592. doi: 10.3390/s22072592 (PMC9002987; doi:10.3390/s22072592)
Supplement: Supplementary file 1 [file sensors-22-02592-s001.zip › sensors-1628627-supplementary.pdf]

Figure S1 shows the chemical structures of caffeine, HBAs, aniline, and resorcinol used in this study. Dotted lines indicate intramolecular H-bond.

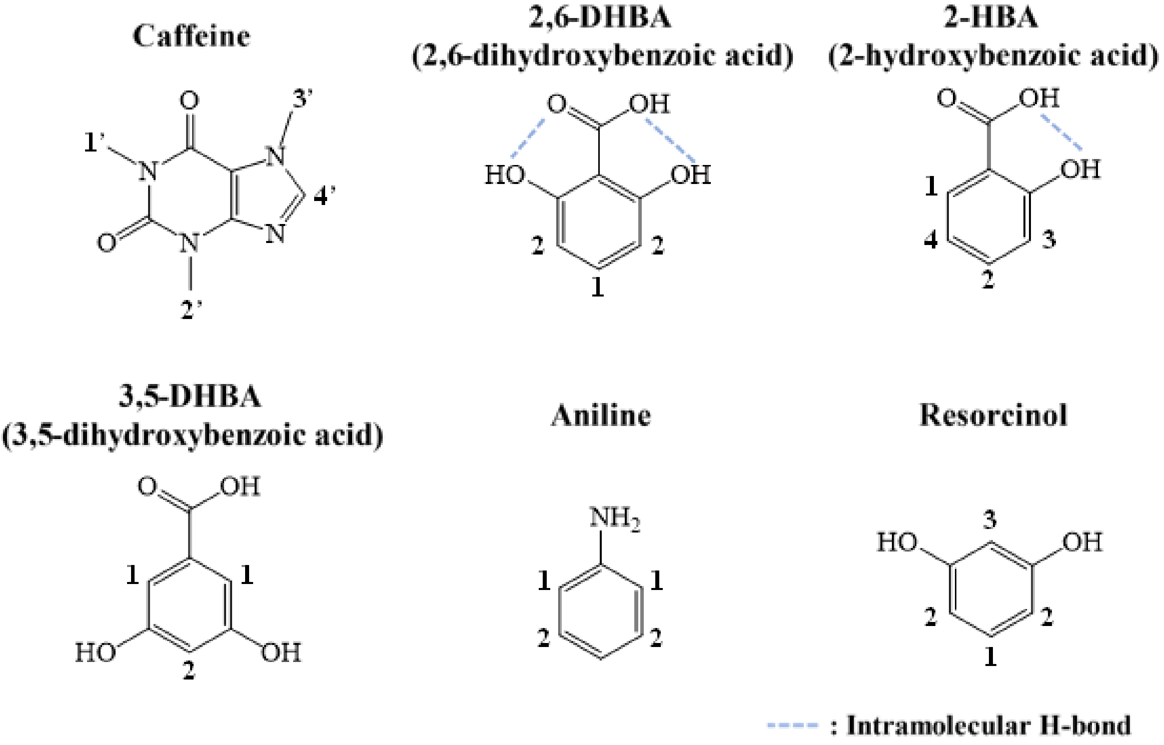

Figure S1. Chemical structures of reagents used in this study.

Figure S2 shows a flowchart of taste sensor measurement. First, the reference solution is measured to obtain V_r. Next, the sample solution is measured and V_s is obtained. In this study, the difference between V_s and V_r is used as the response at the taste sensor. Then, the electrode is washed and this operation is repeated. Reference solution consisting of 30 mM KCl and 0.3 mM tartaric acid. When measuring, the measurement procedure was repeated four times and the mean values and standard deviations (SDs) were calculated from n = 4 (electrode) × 4 (rotation) = 16 electrical response values.

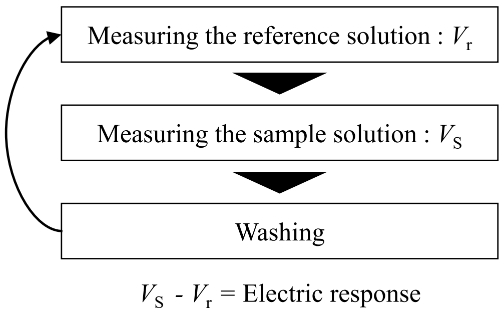

Figure S2. Flowchart of taste sensor measurement.
